# Peer Support for People Living With HIV: A Scoping Review

**DOI:** 10.1177/15248399211049824

**Published:** 2021-10-23

**Authors:** Anita Øgård-Repål, Rigmor C. Berg, Mariann Fossum

**Affiliations:** 1 University of Agder, Grimstad, Aust-Agder, Norway; 2 Norwegian Institute of Public Health, Oslo, Norway; 3 University of Tromsø, Tromsø, Norway

**Keywords:** people living with HIV, chronic disease, peer support, medication adherence, antiretroviral therapy

## Abstract

Peer support for people living with HIV has gained increasing traction and is considered a way to take an active role in self-management. The existing research examining peer support interventions has reported promising evidence of the benefits of peer support. The purpose of our scoping review was to describe research on peer support for people living with HIV. We included 53 studies and sorted them into analytic categories and conducted descriptive analyses. The studies that were published between November/December 2000 and May 2021, had a range of study designs and heterogeneous priority groups, and included 20,657 participants from 16 countries. We identified 43 evaluations of the effect of peer support and 10 evaluations of implementation, process, feasibility, cost of peer support. We also categorized peer support by key functions, finding that the most common key functions were linkage to clinical care and community resources and assistance in daily management, with only one study directly related to chronic care. There is growing research interest in peer support for people living with HIV, particularly in high-income countries and related to the evaluation of effects. The revealed gaps of prioritized functions of peer support have implications for further research. Further focus on interventions addressing secondary prevention related to noncommunicable diseases as part of a care package is recommended to meet people’s needs and preferences and increase self-management related to a chronic lifelong condition.

At the end of 2020, there were an estimated 37.6 million people living with human immunodeficiency virus (PLHIV) worldwide, with approximately 25.4 million undergoing antiretroviral therapy (ART; World Health Organization [WHO], 2021). Although global and national actions have halted and reversed the acquired immunodeficiency syndrome (AIDS) epidemic and reduced HIV incidence overall, HIV infections are on the rise in some countries and regions (WHO, 2021). Furthermore, ART provisions in highly endemic settings, such as sub-Saharan Africa, are challenged due to shortages linked to universal health coverage (UNAIDS, 2020). Thus, HIV remains a public health concern worldwide. The Global Health Sector Strategy on HIV, 2016–2021 (WHO, 2016b), outlines fast-track actions to be implemented as an HIV response to the 2030 Agenda for Sustainable Development (United Nations). These actions must address challenges related to different health care systems and varying health care coverage (such as inconsistent price of medications) across countries. A multisectoral response is outlined as a strategy highlighting the importance of involving the community, particularly PLHIV, for effective delivery of health services (WHO, 2016b).

People from key populations, that is, those at elevated risk of acquiring HIV infection (including sex workers, people who inject drugs, prisoners, transgender people, and men who have sex with men) tend to have less access to ART and health care services (Liamputtong, 2007; Sokol & Fisher, 2016). However, for PLHIV and receiving ART, HIV has become a chronic lifelong condition (CLLC; WHO, 2021). An increasing burden for PLHIV is coinfections such as hepatitis, tuberculosis, and other comorbidities (WHO, 2016b), the most prevalent being noncommunicable diseases and mental health disorders (Brandt, 2009; Parcesepe et al., 2018; WHO, 2016b).

Although the life expectancy for PLHIV has increased dramatically, they continue to face other challenges, such as discrimination, stigma, and self-stigma (Grønningsæter & Hansen, 2018; Pantelic et al., 2019; WHO, 2016b). Since the beginning of the epidemic, HIV infection has been associated with social stigma and prejudice, and it remains one of the most stigmatized diseases in almost every culture, worldwide (Pantelic et al., 2019; Relf et al., 2021). Furthermore, apart from utilizing health care services for HIV medical care, many PLHIV disconnect from society owing to stigma and discrimination (Berg & Ross, 2014; Chaudoir & Fisher, 2018; Relf et al., 2021). The societal prejudice can harm those living with the virus in numerous ways, perhaps most detrimentally, through mental health issues (Chaudoir & Fisher, 2018; Relf et al., 2021).

The range of health challenges indicates the importance of continued strengthening of self-management and involvement of PLHIV in their own health care services. This may contribute to empowerment and a more tailored health care service (Venter et al., 2017). Peer support from the larger HIV community can be important in this regard (Positively UK, 2016) and has been found to reduce stigma (Dunbar et al., 2020). Dennis (2003) defined the concept of peer support as “the giving of assistance and encouragement by an individual considered equal” (p. 323).

Peer support for PLHIV grew out of the 1980s activists’ reaction to combat stigma and discrimination, advocating for better treatment and care. Peer support still forms communities for people experiencing stigma or fear of exposure and ostracization (Positively UK, 2016). After the introduction of ART, peer support has become a tailored, person-centered method to provide linkage and adherence to HIV medical care, as well as support for PLHIV in taking an active role in self-management of their CLLC (Fisher, 2014; WHO, 2016a). Thus, the provision of peer support is one way of involving patients to strengthen supportive resources in health care services and increase self-management (Fisher, 2014). There is increased recognition that peer support complements general health care services and contributes to meeting consumers’ health care needs (Fisher, 2014; Fisher et al., 2018; WHO, 2016a). The Peers for Progress program draws out four key functions of peer support: (1) assistance in daily management, (2) social and emotional support, (3) linkage to clinical care and community resources, and (4) ongoing support related to chronic disease, that is, flexible, accessible support available to patients when the need arises (Fisher, 2014; Fisher et al., 2018).

A systematic review of peer support among “hardly reached individuals,” indicates that peer support may be an effective and preferred way to reach people who do not use ordinary health care services (Sokol & Fisher, 2016). Conversely, a systematic review of nine studies on peer interventions, reported the varying effect of peer support (Genberg et al., 2016). The findings of Genberg et al. (2016) are supported in a recent review on effects of peer-led self-management interventions on ART adherence and patient-reported outcomes, which showed unclear but promising effects (Boucher et al., 2020). Additionally, findings indicate that peer support is flexible enough to be applied to people with different health problems in various settings (Genberg et al., 2016; Simoni et al., 2011; Sokol & Fisher, 2016) and has positive effects, especially in lower middle- and low-income countries (Dave et al., 2019).

Given that existing research examining peer support interventions in several health service areas and among different groups has reported inconsistent evidence of the benefits of peer support (Genberg et al., 2016), there is a need for further research. To date, no review has consolidated existing research or described the scope of the empirical work undertaken on peer support for PLHIV. Therefore, this scoping review aims to document the current status of empirical research on peer support for PLHIV, to describe the characteristics of previous studies through a brief overview, and to summarize key findings from each study category to identify knowledge gaps and offer suggestions for further research.

## Method

### Design

To identify the range of available evidence on the topic, a scoping review was conducted following methodological framework of scoping reviews (Arksey & O’Malley, 2005; Levac et al., 2010; Peters et al., 2017) and is in accordance with the PRISMA (Preferred Reporting Items for Systematic Reviews and Meta-Analyses) extension for scoping reviews (Tricco et al., 2018). Unlike a systematic review on effects of intervention, diagnostic test accuracy or another narrow question, a scoping review has a broader scope, examining the extent, range, and nature of research activity on a specific topic (Peters et al., 2020). The methods, objectives, and inclusion criteria of this scoping review, were specified in advance and documented in a published protocol (CRISTIN ID = 635403).

### Search Strategy for Identification of Studies

Our preliminary searches in the JBI (Joanna Briggs Institute) Database of Systematic Reviews and Implementation Reports and PROSPERO identified relevant reviews and key words. We used population, concept, and context as our search framework because the aim of the scoping reviews implies that the context is not predefined (Booth et al., 2016). Articles published between 1981 and 2021 were searched on eight electronic databases—MEDLINE (OVID), MEDLINE In-Process (OVID), Embase (OVID), CINAHL (EBSCOhost), PsycINFO (OVID), SocINDEX (EBSCOhost), Social Work Abstracts (EBSCOhost), and BASE (Bielefeld Academic Search Engine). Articles published after 1981 were included, as this was the first year when studies on HIV/AIDS were published. The search was conducted in May 2021. Our search strategy incorporated prespecified subject headings and text words in the titles and abstracts, adapted for each database. One of the reviewers (AØR) conducted the search together with an information search specialist/librarian, who was also consulted regarding the search strategy. The search strategy is shown in the Supplemental Material. In collaboration with the information search specialist/librarian, we supplemented the database searches with searches in Google Scholar, the U.K. government website, and CORE (a website that aggregates all open access research outputs from repositories and journals worldwide and makes them available to the public). Additionally, we performed hand searches in the reference lists of the included studies and relevant reviews and forward citation searches through the Web of Science (conducted June 2021).

### Eligibility Criteria

Considering the aim of the review, the main inclusion criterion was that a study used empirical quantitative and/or qualitative research methods to address the topic of peer support among PLHIV. Moreover, both, those receiving and those providing peer support needed to be PLHIV aged 18 years and older. We followed the definition of peer support interventions/programs proposed by Dennis (2003), whereby the provision of assistance and encouragement is from an individual considered equal. Specifically, PLHIV had to use their own experiences to support other PLHIV, through face-to-face interaction. Furthermore, we considered studies ineligible if they included children and youth, focused on primary prevention of HIV or mother-to-child transmission, or described PLHIV support groups. However, when populations or interventions were mixed (e.g., included both adults and youth), a study was included if at least half of the population or intervention met the inclusion criteria or if the results were reported separately for our population and intervention of interest. We enforced no limits regarding settings or publication format but included only publications in English or Scandinavian languages (Norwegian, Swedish, Danish).

### Selection of Literature

We stored retrieved references in an Endnote database, X9 (Thomas Reuters, New York, NY), deleted duplicate entries, and imported references to the web-based software platform, Rayyan (Ouzzani et al., 2016). Using Rayyan, two blinded reviewers independently screened all titles and abstracts according to the inclusion/exclusion criteria. We promoted all relevant publications to full-text, and the two blinded reviewers independently screened the full texts. They attempted to retrieve full texts of any studies that were not available in the public domain, by contacting the main author. Throughout the screening process, we resolved differences in opinions through reexamination of the studies and subsequent discussion. If necessary, a third reviewer decided.

### Data Extraction and Synthesis (Charting Data)

Methodological quality assessment is not a prerequisite for scoping reviews. Therefore, we did not appraise the included studies (Peters et al., 2020). One reviewer (AØR) performed data extraction. Two other reviewers checked for completeness and accuracy of the extracted data. A predesigned and piloted data extraction form was used to ensure standardization and consistency (Peters et al., 2020). We extracted data regarding author, year, study characteristics (e.g., country, study design, sample size), population characteristics (e.g., gender, sexual identity), peer support characteristics (e.g., term for peer support, duration, content, and settings), and main findings/results. We also categorized the interventions based on four key functions of peer support described by Fisher et al. and the Peers for Progress program (Fisher, 2014). Studies with unclear or minimally described intervention characteristics were excluded. We key worded (Clapton et al., 2009) each study using these variables and compiled the data in a single spreadsheet. We grouped them according to their main characteristics and conducted descriptive analyses using frequencies and cross-tabulations. The grouping included sorting the studies into clusters based on how they were observed to be related to each other (Arksey & O’Malley, 2005; Clapton et al., 2009). Similarly, we copied the main ﬁndings of the qualitative studies relevant to peer support, in a Microsoft Word document. The findings are summarized in the data set.

## Results

**Figure 1:**
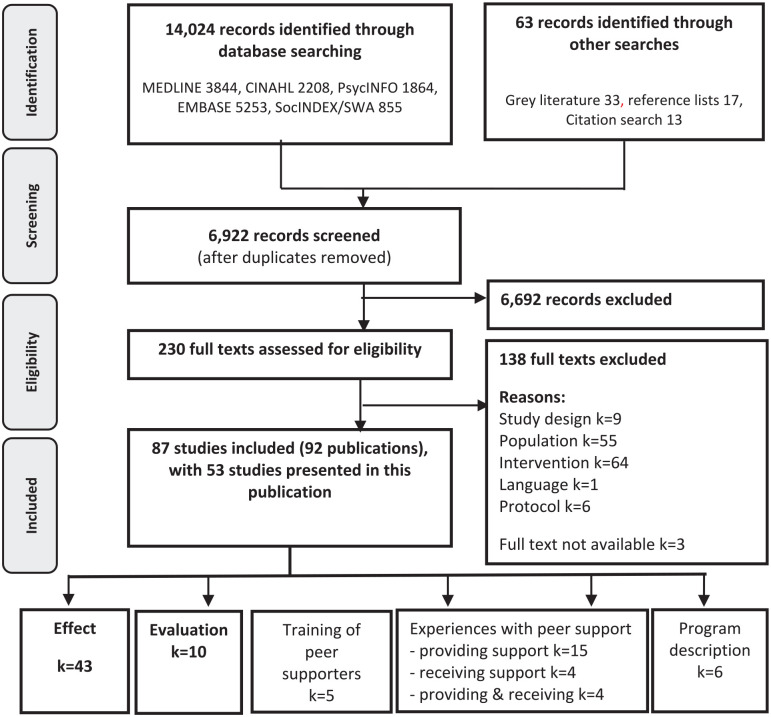
PRISMA (Preferred Reporting Items for Systematic Reviews and Meta-Analyses) Flow Diagram of Literature Reviewing Process

The searches resulted in 6922 individual records, of which 230 were considered potentially relevant (Figure 1). Eighty-seven studies met the inclusion criteria. The high number of included studies and the volume of data made it necessary to separate the results from the two reports. This review addresses all studies that examined the effects of peer support and evaluated implementation, process, feasibility, and cost.

Thus, in this study, we included 53 studies (Table 1).

**Table 1:** Characteristics of the Included Studies (Listed in Alphabetical Order; *N* = 53)

*Study no.*	*Author, year*	*n*	*Country*	*Study design*	*Study focus*
1	Addison et al., 2019	233	USA	Mixed method	Evaluation (implementation)
2	Arem et al., 2011	Ns	Uganda	Mixed method	Evaluation (process)
3	Aung et al., 2021	1,022	Myanmar	Cross-sectional	Effect
4	Been et al., 2020	352	Netherland	Controlled before-after study	Effect
5	Boyd et al., 2005	13	USA	Mixed method	Effect
6	Brashers et al., 2017	98	USA	Mixed method	Effect
7	Broadhead et al., 2012	78	USA	RCT	Effect
8	Broadhead et al., 2002	14	USA	Mixed method	Evaluation (feasibility)
9	Cabral et al., 2018	348	USA	RCT	Effect
10	Campbell, 2008	1,639	USA	Retrospective cohort	Effect
11	Chang et al., 2009	360	Uganda	Retrospective cohort	Effect
12	Chang et al., 2011	970	Uganda	Mixed method	Effect
13	Chang et al., 2010	1,336	Uganda	RCT	Effect
14	Chang et al., 2013	1,416	Uganda	Economic evaluation	Evaluation (economic)
15	Chang et al., 2015	442	Uganda	RCT	Effect
16	Coker et al., 2015	600	Nigeria	RCT	Effect
17	Cunningham et al., 2018	356	USA	RCT	Effect
18	Cuong et al. 2016 ^a^	640	Vietnam	RCT	Effect
19	Dawson-Rose et al., 2020	574	Mozambique	One group posttest only	Effect
20	Deering et al. 2009	20	USA	One-group pre–post evaluation	Effect
21	Derose et al., 2015	482	Honduras	Mixed method	Effect
22	Enriquez et al., 2019	30	USA	Mixed method	Effect
23	Enriquez et al., 2015	20	USA	RCT	Effect
24	Ryerson Espino et al., 2015	Ns	USA	Qualitative	Evaluation (implementation)
25	Fogarty et al., 2001	1,611	USA	Mixed method	Effect
26	Giordano et al., 2016	460	USA	RCT	Effect
27	Graham et al., 2020	60	Kenya	RCT	Effect
28	Graham et al., 2015	40	Kenya	Mixed method	Evaluation (safety, feasibility, and acceptability)
29	Gwadz et al., 2011	342	USA	RCT	Effect
30	Hallum-Montes et al., 2013	30	USA	Qualitative	Evaluation (implementation)
31	Hatcher et al., 2012	483	Kenya	Prospective cohort	Effect
32	Hussein et al., 2020	355	Ethiopia	Pre–post intervention study	Effect
33	Katz et al., 2021	84	South Africa	RCT	Effect
34	Kiweewa et al., 2013	85	Uganda	RCT	Effect
35	Lifson et al., 2017	142	Ethiopia	Mixed method	Effect
36	Liu et al., 2018	367	China	RCT	Effect
37	MacKellar et al., 2021	1,234	Eswatini	One group before and after study	Effect
38	McKirnan et al., 2010	313	USA	RCT	Effect
39	Minick et al., 2018	25	USA	Qualitative	Evaluation (process)
40	Monroe et al., 2017	41	Uganda	Qualitative	Evaluation (process)
41	Pearson et al., 2007	350	Mozambique	RCT	Effect
42	Pokhrel et al., 2018	682	Nepal	Controlled before and after study	Effect
43	Purcell et al., 2007	966	USA	RCT	Effect
44	Reback et al., 2019	139	USA	One group before and after study	Effect
45	Ruiz et al., 2010	240	Spain	RCT	Effect
46	Safren et al., 2011	176	USA	Mixed method	Effect
47	Scarcella et al., 2011	106	Mozambique	Retrospective cohort	Effect
48	Selke et al., 2010	208	Kenya	RCT	Effect
49	Shacham et al., 2018	322	USA	One-group pre/post	Effect
50	Steward et al., 2018	25	South Africa	Qualitative	Evaluation (process)
51	Thomas & Holland, 2018	129	USA	Retrospective chart audit/review	Effect
52	Wewers et al., 2000	15	USA	RCT	Effect
53	Wouters et al., 2014 ^b^	340	South Africa	RCT	Effect

*Note*. RCT = randomized controlled trial.

a This study was reported in multiple publications: see also Cuong et al. 2012 and Van Tam et al. 2012. ^b^This study was reported in multiple publications: see also Masquillier et al. 2014 and Masquillier et al. 2015.

### Characteristics of the Included Studies

The main characteristics of the 53 included studies are presented in Tables 1 and 2. For ease of reporting, each study was given a number. All studies were published in English. The number of publications on the topic of peer support for PLHIV has grown rapidly—from no publications prior to 2000 to only a few publications between 2000 and 2009 (*n* = 9) to 44 publications between 2010 and 2021. The study designs varied, but most were RCTs (*n* = 18) or mixed-method studies (*n* = 12). The study settings varied, but most studies took place in the United States (*n* = 24), while the fewest studies took place in Europe (*n* = 2). The total number of participants in the included studies was 20,657, with most of the studies including both males and females (*n* = 37), but five studies prioritized only males and six prioritized only females. Only four studies included nonbinary gender as the priority population. However, nine studies reported nonbinary gender among participants.

**Table 2:** Summary Characteristics of the Included Studies (*N* = 53)

*Characteristics*	*All studies (N = 53)*	*Effect (n = 43)*	*Evaluation (n = 10)*
Year of publication			
2016–2021	23 (43)	19 (44)	4 (40)
2010–2015	21 (40)	16 (37)	5 (50)
2005–2009	6 (11)	6 (14)	
2000–2004	3 (6)	2 (5)	1 (10)
Country/setting			
Ethiopia	2 (4)	2 (5)	
Kenya	4 (7)	3 (7)	1 (10)
Mozambique	3 (6)	3 (7)	
South Africa	3 (6)	2 (5)	1 (10)
Uganda	8 (15)	5 (12)	3 (30)
USA	24 (45)	19 (44)	5 (50)
Other	9 (17)	9 (20)	
Study design			
RCT	18 (34)	18 (42)	
Mixed method	12 (23)	8 (19)	4 (40)
Other	23 (43)	17 (39)	6 (60)
Gender of participants			
Male	5 (9)	4 (9)	1 (10)
Female	6 (11)	5 (11)	1 (10)
Male and female	37 (70)	30 (70)	7 (70)
Male, female, and trans	4 (8)	4 (9)	
Not stated	1 (2)		1 (10)

*Note*. The “other” countries were China, Eswatini, Ethiopia, Honduras, Myanmar, Nepal, Netherland, Nigeria, Spain, and Vietnam. RCT = randomized controlled trial.

### The Key Functions of Peer Support

Our analysis demonstrates the different roles and key functions (Fisher, 2014) of peer support delivered, in the included studies. The most common key functions of the interventions were linkage to clinical care and community resources (*n* = 41, Studies 1–4, 7–13, 15–20, 22–23, 26–28, 30–35, 37–45, 48–50, 53), followed by assistance in daily management (*n* = 32, Studies 5–6, 9–13, 18–23, 27–28, 35–40, 42–44, 46–48, 50–53) and social and emotional support (*n* = 28, studies 1–9, 11, 15, 19–20, 22–23, 27–28, 33, 35, 37–44, 50). Several peer support interventions have a combination of the described functions. Notably, only one study (44) explicitly focused on ongoing support related to chronic disease. In two studies, the intervention could not be categorized by key functions.

### Terms and Labels

We identified 13 different labels/names for peer supporters. Between 2000 and 2009, the terms “peer,” “peer counselor/advocate/supporter/mentor/health worker,” and “health advocate” were used. Between 2010 and 2021, in addition to the prior labels, a range of new labels appeared: “peer educator/navigator/worker/facilitator/case manager/caregiver/adherence supporter/interventionist,” “community health worker,” “support worker,” and “community care coordinator.” All terms represent PLHIV serving as peers. The most frequently used labels across all included studies were “peer” (*n* = 10), “peer counselor” (*n* = 7), and “peer navigator” (*n* = 6).

### Categories of Studies

We categorized studies by objective/aim (see Figure 1). When a study fit into more than one category, we placed it in the category that most closely matched the overall objective of the article. This review included two study categories: studies evaluating effects of peer support interventions (*n* = 43) and studies evaluating their implementation, process, feasibility, and cost (*n* = 10). We note that six larger projects on peer support had two or more related publications that examined the intervention: all six projects had at least one publication on the effects of peer support; four projects conducted a process evaluation, and two projects included a program description.

#### Studies About Effectiveness of Peer Support

Of the 43 studies with a main focus on the effectiveness of a peer support intervention (Studies 3–7, 9–13, 15–23, 25–27, 29, 31–38, 41–49, 51–53), most were published within the past 10 years (81%), were set in the United States (44%) and Uganda (12%), and were RCTs (42%) and used mixed methods (19%; Table 3). Only two studies were conducted in Europe (Netherlands and Spain: Studies 4 and 45). In total, 18,833 participants were included in the experimental studies at baseline. Of the 30 effectiveness studies that had a comparison group, 21 of these groups received ordinary health care services.

**Table 3:** Characteristics of Effect studies Related to PICO (Population, Intervention, Comparison, and Outcome) in Alphabetic Order (*N* = 43)

*Study (country)*	*Population*	*Intervention*	*Comparison*	*Outcome domains*
Aung et al., 2021 (Myanmar)	*N* = 1,022, female and male	A counselor met with the patient over 1–3 pre-ART sessions. Based on counselor availability, the patient may or may not have retained the same counselor through all sessions. Once PC or SC counselling was complete, patients initiated ART.	Usual care	HIV knowledge Enacted and internalized stigma ART-nonadherence Barriers to care Social support Attitudes regarding counselling
Been et al., 2020 (Netherland)	*N* = 352, Migrants living with HIV	*RO*tterdam *AD*herence (ROAD) project. Four existing interventions that could potentially improve treatment adherence in MLWH. The fourth of these interventions was peer support by MLWH, whereby individuals have increased access to emotional support, informational support, and appraisal support.	No comparison group	Social support Internalized stigma Adherence Anxiety and depression
Boyd et al., 2005 (USA)	*N* = 13, Women, substance use	Provide emotional and informational support to change their substance abuse patterns and problem-focused coping strategies. Motivation enhancement therapy (MET). The intervention was implemented over an 8- to 12-week period.	No stated	Substance use
Brashers et al., 2017 (USA)	*N* = 98, female and male, newly diagnosed	“Living with HIV/AIDS: Taking Control”. Educate people newly infected with HIV about the disease, treatments and resources available. The group had one session per week for 6 weeks.	Usual care	Illness uncertainty Social support Level of depression symptoms Self-advocacy
Broadhead et al., 2012 (USA)	*N* =78, female and male, IDUs	Intervention (PDI) model rewards drug users who recruit their own peers. Participants play two roles: promoters of adherence and recipients of an advocate’s efforts.	Usual care	Enrollment in and utilization of primary care services for HIV Drug- and sex-related risk behavior Adherence skills
Cabral et al., 2018 (USA)	*N* = 348, female and male, people of color	Intervention based on the social support framework. The peer addressed four domains: (1) informational support, (2) instrumental support, (3) emotional support, and (4) affiliation support.	Usual care	Retention in care Viral suppression
Campbell, 2008 (USA)	*N* = 1,639, female and male	The Peer Mentor Program to establish and develop an effective mentor system to assist HIV-positive patients as they access care and services in this urban HIV-specific clinic for the first time.	Usual care	Retention in HIV care Viral load CD4 counts
Chang et al., 2009 (Uganda)	*N* = 360, female and male, low socioeconomic status	The Reach Out care model: A patient-led, holistic approach to AIDS care with the provision of comprehensive health and social services.	No stated	ART adherence Viral load CD4 counts Immunologic
Chang et al., 2011 (Uganda)	*N* = 970, female and male	Peer health workers (PHWs) randomized to the mHealth Arm send a text message reporting adherence and clinical data back to a centralized database after home visit. PHWs in the mHealth Arm were encouraged to call a RHSP mobile phone or toll-free warmline with questions or concerns. Clinic staff receiving PHW texts and calls could opt to provide care instructions to PHWs, send a higher level care provider to the patient, or arrange transport to health care facilities.	The comparison group consisted of PHWs who did not receive the mHealth intervention.	ART adherence Viral load Lost to follow-up Mortality
Chang et al., 2010 (Uganda)	*N* = 1,336, female and male, on ART	Task shifting with PHW. ART provided through a mobile clinic program. A PHW intervention delivers additional support.	Usual care	ART adherence Viral load
Chang et al., 2015 (Uganda)	*N* = 442, female and male	Peer support structured home visits to promote clinic attendance and preventive care intervention use or standard of care. Peers visited each participant monthly.	Usual care	ART initiation Retention in care BCP preventive care use Risky sexual behaviors
Coker et al., 2015 (Nigeria)	*N* = 600, female and male	Participants were randomized into one of three intervention arms: a standard-of-care arm; a second arm included daily reminders via alarm, follow-up calls from peer educators, and adherence support by a home-based treatment partner; and a third arm included second-arm activities plus home visits by peer educators	Usual care	ART adherence Viral load CD4 counts
Cunningham et al., 2018 (USA)	*N* = 356, men or transgender women released from a large municipal jail system	LINK LA. A peer navigation intervention. The intervention group participated in a 24-week peer navigation intervention. Trained peer navigators counselled participants on goal setting and problem solving.	Usual care	ART use and adherence Viral load Linkage to care Retention in HIV care Retention/adherence knowledge Physical and mental health
Cuong et al., 2016 (Vietnam)	*N* = 640, female and male, treatment-naïve	Peer supporters visited their homes twice a week during the first 2 months, followed by once a week afterward. The peer supporters asked the patients about their general well-being, social–psychological problems, OI symptoms, adverse drug reactions, and adherence and conducted a pill count. They also performed some home care and referred patients to hospitals.	Usual care	ART initiation Viral load CD4 count Mortality rate Causes of death and risk factors
Dawson-Rose et al. 2020 (Mozambique)	*N* = 574, female and male	Peer educators helped clients to explore ways to disclose to partners. They provided emotional support. They provided information on the four identified and UNAIDS-approved steps of disclosure to HIV positive clients.	No comparison group	HIV disclosure
Deering et al., 2009 (USA)	*N* = 20, female sex workers who use illicit substances	The PDI intervention consisted of four key elements: weekly peer support meetings, capacity training for women to become health advocates (‘‘buddies’’) to one another, a peer outreach service, and drop-in onsite nursing service.	No comparison group	ART adherence Viral load
Derose et al., 2015 (Honduras)	*N* = 482, female and male, on ART	A simplified version of visual aids and a reference technical manual based on a nutrition education curriculum were developed for peer counselors, supported by educational materials.	No comparison group	Food insecurity Nutritional knowledge/dietary intake Nutritional status
Enriquez et al., 2019 (USA)	*N* = 30, female and male, nonadherence to ART medications	The Peers Keep It Real intervention program consisted of seven individual sessions facilitated by a peer interventionist. It occurred at the health care setting where the participant would obtain his or her HIV care and were scheduled on the same day as appointments with HIV medical care providers.	Usual care	ART adherence Viral load
Enriquez et al.,2015 (USA)	*N* = 20, female and male, nonadherence to ART medications	“READY”. The peer support is based on the readiness stage of the wellness motivation theory and framed in understanding the process of initiating and maintaining healthful behavior change. Tailored to enhance its cultural relevance for a target population living day-to-day in a culture of HIV.	A graduate health psychology student facilitated education	ART adherence Viral load
Fogarty et al., 2001 (USA)	*N* = 1,611, women and at-risk women	The intervention included support groups and one-on-one contact with peer advocates tailored to client needs. The strategies included group support, peer advocacy, multiple sessions, tailored educational messages and theory.	Usual care	Demographic and risk data Behavioral outcomes Self-efficacy Perceived advantages
Giordano et al., 2016 (USA)	*N* = 460, female and male, hospitalized HIV-infected patients who had never been in outpatient HIV care, had been poorly retained in care, or had detectable HIV viral load	The intervention MAPPS. The intervention is delivered during 2 in-person sessions in the hospital, followed by 5 telephone calls after discharge over the next 10 weeks. The intervention focused on mentors serving as role models for successfully managing HIV infection and for encouraging active self-management.	The control intervention was delivered as the MAPPS intervention but with a different goal and approach.	Retention in care Viral load
Graham et al., 2020 (Kenya)	*N* = 60, MSM	The Shikamana intervention. The intervention combines modified Next-Step Counseling by trained providers, support from a trained peer navigator, and tailored use of SMS messaging, phone calls, and discrete pill carriers.	Usual care and an invitation to attend a monthly support group	ART adherence Viral load
Gwadz et al., 2011 (USA)	*N* = 342, female and male, people of color	The intervention’s mechanisms of action were grounded in the theory of normative regulation, as well as motivational interviewing and social–cognitive theory. The intervention included 6 hours of structured, facilitated sessions plus the opportunity to educate up to 3 peers about AIDS clinical trials.	A time- and attention-matched health education intervention.	Participation in screening for AIDS clinical trials
Hatcher et al., 2012 (Kenya)	*N* = 483, female and male, not previously enrolled in HIV care or treatment	HIV counseling and testing (HCT) was offered in accordance with the Kenyan national guidelines. Newly diagnosed clients were invited to receive a follow-up home visit by a trained PLWHA navigator. Following the campaign, PLWHA navigators attempted to conduct home visits with all persons providing locator information to offer support for enrolling into HIV care.	No comparison group	Linkage to care
Hussein et al., 2020 (Ethiopia)	*N* = 355, female and male	Peer education not specified.	No comparison group	ART adherence
Katz et al., 2021 (South Africa)	*N* = 84, female and male who delay or discontinue ART	The theory of triadic influence (TTI) designed to address individual-, social-, and structural-level barriers to ART initiation: (1) individual-level factors by building the knowledge base and trust of treatment, while promoting self-efficacy and effective coping strategies; (2) social-level factors through social interactive processes that address HIV-related stigma and the need for disclosure; and (3) structural-level factors through facilitating engagement with clinic providers.	Usual care	General health perceptions Depression, anxiety, and somatic complaints Social support Stigma and disclosure concerns Barriers to ART
Kiweewa et al., 2013 (Uganda)	*N* = 85, female on ART	The model evaluated the effect of a task-shifting model in which ART nurses managed most follow-up visits at longer intervals between visits, and patients were supported by peer counselors and home visits, if indicated.	Usual care	Initiated ART Viral load CD4 count
Lifson et al., 2017 (Ethiopia)	*N* = 142, female and male, newly enrolled in HIV clinical care	The CHSWs provided HIV and health education and counseling/social support, as well as facilitated communication with the HIV clinics. The CHSWs visited clients 1 to 4 times/month to provide the following: (1) education on HIV treatment, nutrition, and other health-promoting behaviors; (2) counseling and social support; (3) facilitated communication with the nurse from the HIV clinic; and (4) referrals as needed to community organizations	No comparison group	Retention in care HIV knowledge, physical and mental quality of life Internalized stigma Perceived social support
Liu et al., 2018 (China)	*N* = 367, newly diagnosed, MSM	The peer counseling manual was based on an adapted information–motivation–behavioral skills (IMB) model. The peer counseling session involved a 60-minute one-on-one discussion focusing on topics regarding specific high-risk behavior modification	Usual care	High-risk behavior change Quality of life HIV stigma Self-efficacy Hospital anxiety and depression
MacKellar et al., 2021 (Eswatini)	*N* = 1,234, female and male	Community-based HIV testing, mobile HIV care, and peer delivered, linkage case management program (CommLink). The CommLink package of linkage services included, for example, peer-delivered counseling, at least two additional (three total) face-to-face HIV counseling and psychosocial-support sessions.	No comparison group	Barriers to HIV care
McKirnan et al., 2010 (USA)	*N* = 313, MSM	The Treatment Advocacy Program (TAP). The intervention consisted of four 60- to 90-minute individual counseling sessions, 3-month “check-in” telephone calls, and 6- and 12-month coping follow-up counseling sessions.	Usual care	Sexual risk behavior
Pearson et al., 2007 (Mozambique)	*N* = 350, female and male, initiating HAART	Participants received 6 weeks (Monday through Friday; 30 daily visits) of peer-delivered modified directly observed therapy (mDOT). Peers provided education about treatment and adherence and sought to identify and mitigate adherence barriers.	Usual care	Adherence to HAART CD4 counts
Pokhrel et al., 2018 (Nepal)	*N* = 682, female and male, on ART	The intervention encompassed home-based psychosocial support and peer counseling, adherence support, basic health care, and referral services. The support team comprised a community health worker, a trained HIV-positive person, and a social worker.	Usual care	ART adherence Depressive symptoms Anxiety Stress Substance use
Purcell et al., 2007 (USA)	*N* = 966, female, male and transgender, IDUs	The INSPIRE project. The peer-mentoring intervention (PMI, the intervention condition) was developed based on a combination of theories and concepts. INSPIRE integrated key concepts of empowerment theory into an HIV prevention intervention.	The control condition: eight small-group video-and-discussion sessions on topics relevant to participants’ lives.	Substance use Sexual risk behavior Retention Adherence
Reback et al., 2019 (USA)	*N* = 139, transgender women	The Alexis Project was a combined peer health navigation (PHN) and contingency management (CM) intervention that targeted HIV milestones associated with advancement along the HIV care continuum.	No comparison group	Linkage to care Retention CD4 counts HIV medication pick-up
Ruiz et al., 2010 (Spain)	*N* = 240, female and male, on ART	The intervention group was treated by a “peer.” Patients in both groups received a psychoeducational intervention to increase their adherence to ART. In addition to the baseline visit, patients were seen at Weeks 8, 16, and 24. The intervention visits were scheduled to coincide with routine hospital visits to facilitate attendance by the patients (adherence to the intervention).	Group A was treated by a health professional (physician or pharmacist with extensive knowledge about HIV)	ART adherence Viral load Psychological distress
Safren et al., 2011 (USA)	*N* = 176, MSM	A peer-driven IMB. The intervention included five visits with an HIV-infected MSM peer interventionist over the course of approximately 3 months, which included one “intake” visit, and four “intervention” visits. These were followed by four follow-up “booster” visits at 3, 6, 9, and 12 months postintervention. The intervention was delivered in the clinic setting.	Medical social workers as interventionists who had specific HIV-risk inclusion/exclusion criteria and intensive study procedures	The feasibility of delivering an HIV sexual risk reduction counseling program in the context of primary HIV care Acceptability of the intervention Transmission risk behavior
Scarcella et al., 2011 (Mozambique)	*N* = 106, female and male, BMI< 18.5, presence of the wasting syndrome, TB coinfection, insufficient access to food, CD4 count < 200	The DREAM program. The program is characterized by provision of HAART, clinical and laboratory monitoring, peer-to-peer health and nutritional education, and food supplementation. The activists become peer-to-peer health and nutrition educators, and they are particularly involved in supporting adherence to the therapy and promoting food hygiene and a balanced diet.	No comparison group	BMI Hemoglobin Viral load CD4 count Dietary intake
Selke et al., 2010 (Kenya)	*N* = 208, female and male, on ART	The intervention group received monthly personal digital assistant-supported home assessments by PLHWA at clinic appointments every 3 months.	Usual care	Viral load CD4 count Stability of ART regimen Opportunistic infections Pregnancies Number of clinic visits
Shacham et al., 2018 (USA)	N=322, female, male and transgender	The Barrier Elimination and Care Navigation (BEACON) Project Evaluation. Participants enrolled in a community- and clinic-based intervention that included intensive case management, access to a community nurse and peer navigator, and emergency stabilization funds.	No comparison group	Quality of life Biomedical markers of HIV Retention in care
Thomas and Holland, 2018 (USA)	*N* = 129, female	The intervention consisted of peer mentors providing information about cervical cancer screening and assisted with scheduling a gynecological visit. Peer mentors educate, support, and provide linkage to health care services to persons living with HIV.	Usual care	Cervical cancer screening uptake
Wewers et al., 2000 (USA)	*N* = 15, female and male, self-reported smoking	Smoking cessation intervention. The intervention was based on the Agency for Health Care Policy and Research Smoking Cessation Clinical Practice Guideline and was delivered by an ex-smoker who was HIV positive. The intervention was delivered primarily by a peer.	They were mailed the same written materials as the intervention group including a strong message to quit smoking.	Abstinence rates
Wouters et al., 2014 (South Africa)	*N* = 340, female and male, on ART	The peer intervention was developed based on the family functioning framework. It focused on family dynamics in community-based peer adherence support. Peer adherence support comprised biweekly visits by a trained community-based peer. he peers performed a wide range of adherence counseling tasks.	Usual care	CD4 counts ART adherence

*Note*. ART = antiretroviral therapy; PC = peer counselor; SC = standard counselor; MLWH = migrants living with HIV; IDU = injection drug user; PDI = peer-driven intervention; RHSP = Rakai Health Sciences Program; BCP = basic care package; OI = opportunistic infection; UNAIDS = Joint United Nations Programme on HIV/AIDS; MAPPS = Mentor Approach for Promoting Patients’ Self-Care; MSM = men who have sex with men; SMS = short message service; PLWHA = people living with HIV/AIDS; CHSW = community health support worker; HAART = highly active antiretroviral therapy; INSPIRE = Interventions for Seropositive Injectors-Research and Evaluation; BMI = body mass index; TB = tuberculosis; DREAM = Drug Resources Enhancement against AIDS and Malnutrition.

Although, the priority population of the effectiveness studies was diverse, the studies mainly included female and male participants living in settings associated with social factors that created barriers to accessing effective and affordable HIV health care services. Five studies included only women (Studies 5, 20, 25, 34, 51), four included people who inject drugs (Studies 5, 7, 20, 43), four recruited men who have sex with men (Studies 27, 36, 38, 46), and two U.S. studies specifically recruited people of color (Studies 9, 29).

About the chosen theoretical framework, the interventions differed. Most interventions were based on diverse frameworks, such as social cognitive theory (Studies 17, 19, 23, 29), several social support frameworks (Studies 9, 7, 42, 43), stress, and coping models (Study 5). Several interventions were based on the information, motivation, and behavioral skills model (*n* = 7; Studies 15, 21, 26, 27, 36, 41, 46). Eighteen studies did not report a theoretical framework.

ART initiation and/or adherence (19 studies), viral load (16 studies), and cluster of differentiation 4 counts (CD4; 8 studies) were the most frequently measured outcomes in the included studies related to effectiveness. Other measured outcomes were retention in care, adherence to medical care, mental health, sexual behaviors among PLHIV, quality of life, and stigma. With respect to findings, most studies measuring ART initiation and/or adherence found a positive effect, but not all. One study measured HIV stigma and three others assessed internalized stigma. However, only two studies reported their results, which found decreased negative feelings and enacted/internalized stigma. Similarly, the results for the other outcomes varied. It is important to bear in mind that the populations, content of peer support, comparisons, and length of follow-up varied.

#### Evaluation Studies

The other evaluation studies focused on implementation (Studies 1, 24, 30), process (Studies 2, 39, 40, 50), feasibility (Studies 8, 28), and cost (Study 14; Table 1). They included 1824 male and female participants from the United States (*n* = 5), Uganda (*n* = 3), Kenya (*n* = 1), and South Africa (*n* = 1).

##### Implementation

The three studies on implementation were qualitative (n = 2) and mixed methods (n = 1) design. They described barriers, challenges, and strategies related to the implementation of peer support interventions as a link to care for PLHIV. One study concluded that the intervention was best suited to newly diagnosed patients (Study 1), while the other two reasoned that the specific settings affected the implementation of peer-based programs and offered considerations on the quality of the training and support of peers and their integration in the delivery of health services (Studies 24, 30).

##### Process

There were four process evaluations of qualitative (*n* = 3) and mixed-methods (n = 1) design. All sought to understand the underlying mechanisms of the intervention results: gain insight into lack of effect (Study 2), clarify positive effects (Study 50), examine how to improve the intervention (Study 39), and investigate why and how peer supporters improved client engagement in care (Study 40).

##### Feasibility

Both studies on feasibility had a mixed methods design. One was related to the willingness and ability of persons who inject drugs to help each other. Findings indicated a high level of willingness and that the peer support intervention increased their adherence to care (Study 8). Another study, which examined the engagement of Kenyan men who have sex with men, concluded that the peer support intervention was feasible and acceptable to the participants (28).

##### Cost

The economic evaluation analyzed and compared the costs of a peer health worker intervention and a phone peer support intervention (Study 14). While both interventions were evaluated as potentially cost-effective, the threshold analysis suggested that the peer health worker intervention was potentially most cost-effective if it was able to avert 1.5 patients every year from switching to second-line ART.

## Discussion

Our scoping review, aimed to describe the characteristics and results of evaluation research on peer support for PLHIV, identified 53 studies, all published since 2000. Research on peer support for PLHIV has grown rapidly over the past decade. This may reflect the increased life expectancy of PLHIV following the introduction of ART and, hence, peer support becoming a more integrated part of health care services.

### Different Populations and Intervention Characteristics

The 53 studies demonstrated heterogeneity of populations, intervention characteristics, outcomes, and settings investigated in peer support programs. Most studies had both females and males as the priority population for peer support. Other priority groups included people who inject drugs, men who have sex with men, people of color, and individuals with little disposable income, which uncovered a varied priority population. Consistent with the aim of health promotion strategies and the Global Health Sector Strategy on HIV 2016–2021 (WHO, 1986, 2016b), it seems these investigations represent a diversity of needs of PLHIV. However, it is also worth mentioning the low number of studies that included nonbinary genders. This was true despite these individuals being at increased risk of acquiring HIV infection compared with the general population (UNAIDS, 2020). The geographical aspect is noteworthy. A large proportion of the included studies were conducted in low-resource settings and in the U.S. regions heavily affected by the HIV epidemic, while only two were conducted in Europe. This suggests that there is limited interest in this intervention among researchers in Europe.

Furthermore, the most common key intervention function, used in 41 of the interventions, was linked to care and community resources, which is important to strengthen the health care workforce related to HIV. From this perspective, peer support attempts to respond to the needs of PLHIV in priority settings. The key functions “assistance in daily management” and “linkage to care and community resources” have the flexibility to engage those living with HIV in the process of planning peer support. This involvement ensures that peer support fits the priority population. A setting-specific approach acknowledges that low-resource and high-resource settings have different needs, which is evident in the context of studies.

### A Reflection on Measured Outcomes

Biological markers, such as viral load, CD4 counts, and adherence to ART, were the most frequently measured outcomes in the included studies. A recent systematic review detailed findings on these outcomes (Berg et al., 2021). Only four of our studies measured stigma as the primary outcome. This is despite stigma being a known barrier to HIV treatment and care (Relf et al., 2021), with studies showing that it affects the degree of disclosure, followed by decreased social support and health-seeking behavior (Smith et al., 2008).

It is important to measure the effect of peer support on perceived stigma. Research shows that interventions that increased linkages to care and community resources, as well as social and emotional support, were able to facilitate improvements in mental health status and had the potential to enable those living with HIV to overcome the effects of anticipated and internalized stigma (Garrido-Hernansaiz & Alonso-Tapia, 2017). Thus, social support from peers may be a resource when people experience stress in response to stigma (Dulin et al., 2018; Dunbar et al., 2020; Earnshaw et al., 2015).

We also found a need to clarify the support needed by PLHIV as individuals living with a CLLC. Although anticipated and/or experienced stigma might affect their general efforts to seek support, the included studies indicate that meeting a peer supporter may contribute to social support. However, few studies have measured whether and how peer support affects aspects of mental health and quality of life as primary outcomes, despite the high rates of documented mental health disorders among PLHIV (Brandt, 2009; Parcesepe et al., 2018). This could be related to the scant amount of peer support related to chronic diseases as a key function, according to the definition of ongoing chronic support by the Peers for Progress program (Fisher, 2014; Fisher et al., 2018). Despite the large number of studies that support self-management, social and emotional support, and linkage to HIV care, few studies have reported peer support as a long term, flexible outreach program.

### What Defines Peers?

We found little uniformity in terms of both the terminology and practice of peer support. We identified 13 different labels/names for peer supporters, with the most frequently used label being “peer’. This is somewhat surprising considering our narrow inclusion criteria. In their review of “Peer Interventions to Promote Health: Conceptual Considerations,” Simoni et al. (2011) proposed the term “peer” as standard terminology with an extended definition consisting of four elements: (1) peers share key personal characteristics, circumstances or experiences with the priority group; (2) the benefits of a peer intervention derive largely from their status as peers; (3) peers do not need professional training; and (4) peers function according to a specific role. The first element coincides with a definition proposed by Dennis (2003). Still, Simoni et al. (2011) used a clearer conceptualization to distinguish peer work interventions from work by others involved in services. In this terminology, the definition of Dennis (2003) might have a wider reach than Simoni’s, although Simoni’s definition is more focused on peer roles. The variation of labels discovered across the included studies in this review may suggest that different labels fit different interventions. We categorized the key functions of peer support and found that three key functions were part of most interventions—only one focused on ongoing support related to chronic disease and two studies lacked information on key functions. It is necessary to understand the characteristics and primary key functions of peer supporters. When the intervention characteristics are insufficiently described or poorly reported, and the intervention subsequently appears to exist in many variants under different labels, it becomes harder to understand what is meant when “peer support” and similar terms are used.

### Agreements and Disagreements With Other Studies or Reviews

Several reviews on peer support interventions for PLHIV have been conducted. While focusing on separate aspects, these largely mirror our findings. First, Simoni et al. (2011) conducted a systematic review to investigate the efficacy of different types of peer support in HIV/AIDS patients. The review resolved some effects of peer interventions, but heterogeneity in populations and outcomes affected the ability to draw conclusions. These authors and authors of a review published a decade later (Berg et al., 2021) state that additional, carefully designed studies are required to investigate the effectiveness of peers and the conditions that need to be present to ensure successful interventions. This reflects our finding that various intervention characteristics, settings, and outcomes challenge the ability to compare interventions. Genberg et al. (2016) conducted a systematic review of peer interventions to improve engagement in care, indicating that peers had a mixed impact on ART adherence, viral suppression, and mortality. Although peer interventions had a positive effect on linkage to and retention in care, a limited number of studies have measured these outcomes. Decroo et al. (2012) published a review that examined whether expert patients were an untapped resource of ART provision in sub-Saharan Africa. Findings indicated that PLHIV can serve as a resource in the provision of ART in this region, which is promising in this high-epidemic area. Notably, we have identified no reviews on the implementation of peer support, process evaluation, or cost analysis.

### Implications

The increased number of publications on peer support for PLHIV over the last decade has shown a growing interest in this topic. Despite this, we recognize the need for more studies in Europe and sub-Saharan Africa. Only two studies were from Europe, and less than 40% of the included studies were conducted in sub-Saharan Africa, which is a high-epidemic area of HIV, identified by the WHO as a priority population (WHO, 2016b). There have been no studies from Russia, which is one of the few countries with growing HIV incidence rates. Areas such as sub-Saharan Africa and Russia are in need of fast-track action (WHO, 2016b), and research evidence from other areas with comparable populations can be transferred to these. However, there will be a lack of setting-specific knowledge. A handful of forthcoming studies on peer support for PLHIV are registered at ClincalTrials.gov. They mostly relate to the prevention of HIV, which is promising; however, few prioritize the population in sub-Saharan Africa.

Our results show that the most common key characteristics of peer support are linkage to care and community resources, assistance in daily management, and social and emotional support. These are appropriate for the priority population and the settings of the existing interventions and can, arguably, have an impact on stigma, mental health, and quality of life. Our results suggest a broader scope when the effects and experiences of peer support are measured in relation to living with HIV, knowing that new needs arise throughout life when living with a CLLC (Fisher, 2014; Fisher et al., 2018). As noted, our results align with existing global strategies and guidelines, and have relevance for policy makers and health care providers. As indicated by other reviews (Berg et al., 2021), the results support that peer support can help shoulder existing services. The Global Health Sector Strategy on HIV 2016–2020 recommends an integrated care package designed to meet people’s needs and preferences and increase self-management related to CLLC. Hence, peer support is a type of care package that can meet the various needs of PLHIV. Further focus on interventions addressing secondary prevention related to noncommunicable diseases as part of this package is recommended.

Because of its broad aim and inclusion of studies, this review is summative in nature and provides an opportunity for detailed analysis of effect studies in particular. Our results further demonstrate the scarcity of studies on the implementation, process, and cost analyses. These are important perspectives for researchers and health care entities in consideration of improvement of peer support services.

### Strengths and Limitations

The systematic approach regarding searches, selection, and data extraction is the main strength of our scoping review, although a limitation of the review is the absence of studies in languages other than Scandinavian and English. Our framework helped us to be consistent in the approach, and the data analyses made it possible to identify and maintain consistency for all categories. The broad scope of this review, along with the large number of included studies with diverse findings, limited the opportunity to draw firm conclusions. This review provides a comprehensive overview of the research field on the evaluation of peer support for PLHIV. A main limitation was that the included studies had several labels for peer supporters that were previously unknown to the reviewers. It is possible that this could have affected the search strategy, and we might have missed some relevant studies.

## Conclusions

This scoping review documented an increased research interest in peer support for PLHIV, although it revealed gaps in *where* the research was conducted, *outcomes* measured, and prioritized function of peer support related to chronic care. With about 25.4 million people accessing ART, the need for support related to retention in care and chronic care is increasing. The gaps in the prioritized functions of peer support have implications for further research. The flexibility of the peer support role related to settings, health outcomes, and populations appears to complement health care services with regard to the different needs of PLHIV.

## Supplemental Material

sj-docx-1-hpp-10.1177_15248399211049824 – Supplemental material for Peer Support for People Living With HIV: A Scoping Review Click here for additional data file. Supplemental material, sj-docx-1-hpp-10.1177_15248399211049824 for Peer Support for People Living With HIV: A Scoping Review by Anita Øgård-Repål, Rigmor C. Berg and Mariann Fossum in Health Promotion Practice

sj-docx-2-hpp-10.1177_15248399211049824 – Supplemental material for Peer Support for People Living With HIV: A Scoping Review Click here for additional data file. Supplemental material, sj-docx-2-hpp-10.1177_15248399211049824 for Peer Support for People Living With HIV: A Scoping Review by Anita Øgård-Repål, Rigmor C. Berg and Mariann Fossum in Health Promotion Practice
