# Supplementary material for: Peer Support for People Living With HIV: A Scoping Review
Source: Health Promot Pract. 2021 Oct 23;24(1):172–90. doi: 10.1177/15248399211049824 (PMC9806454; doi:10.1177/15248399211049824)
Supplement: sj-docx-2-hpp-10.1177_15248399211049824 – Supplemental material for Peer Support for People Living With HIV: A Scoping Review [file sj-docx-2-hpp-10.1177_15248399211049824.docx]

**Search strategy**

**MEDLINE, EMBASE and APA PsycInfo (Ovid)**

Database: Embase <1980 to 2021 Week 20>, Ovid MEDLINE(R) ALL <1946 to May 21, 2021>, APA PsycInfo <1806 to May Week 3 2021>. [Search Strategy: link](https://ovidsp.ovid.com/ovidweb.cgi?T=JS&NEWS=N&PAGE=main&SHAREDSEARCHID=4ujvMuXd8DWXJ6UcoxPVXTfrNn29sUE47fyKy9wzeL0KJF3ZzFPNs39emsybEpxAr) results based on search date: 23.05.2021

--------------------------------------------------------------------------------

1 ((hiv or aids) adj6 (patient* or people* or person* or client* or living or men or women or woman or female* or adult* or service* or support* or positiv* or care or caring or affect*)).ti,ab. (470967)

2 (peer or peers).hw. (134476)

3 peer*.ti,ab. (332044)

4 (lay adj3 (people* or patient* or client*)).ti,ab. (5993)

5 (patient* adj2 expert*).ti,ab. (7232)

6 2 or 3 or 4 or 5 (392228)

7 1 and 6 (8403)

8 exp HIV Infections/ or exp Human immunodeficiency virus infection/ or hiv/ (794523)

9 exp Anti-Retroviral Agents/ or acquired immune deficiency syndrome/ or Acquired Immunodeficiency Syndrome/ or aids/ (482469)

10 8 or 9 (917056)

11 (peer* adj6 (group* or support* or couns* or service* or provide* or care* or mentor* or tutor* or educat* or led)).ti,ab. (81375)

12 2 or 11 (184850)

13 10 and 12 (6210)

14 7 or 13 (10836)

15 ((hiv* or aids) and peer*).ti. (1417)

16 14 or 15 (10968)

17 limit 16 to yr="1981 -Current" (10961)

18 limit 17 to yr="1981 - 2013" (5477)

19 remove duplicates from 18 (3222)

20 limit 17 to yr="2014 -Current" (5484)

21 remove duplicates from 20 (3222)

22 21 or 19 (6444)

**Notes on search syntax**

- Adj6 = N5 (EBSCOhost), adjacency. . The ADJ3 operator finds terms in any order with two words (or fewer) between them. The ADJ4 operator finds terms in any order and with three words (or fewer) between them, and so on
- Field codes used
  - ti,ab = words from title, abstract (text words)
  - .hw = single word from a subject heading word/or part of a subject heading phrase like “peer tutoring”
  - / exact subject headings- search 8 and 9 – subject headings used in the three databases
  - Exp / exact subject headings including narrowing terms – search 8 and 9 subject headings used in the three databases

| **Notes on the searches** |  | Search string | Results |
| --- | --- | --- | --- |
| Population HIV/AIDS  Word from title or abstract | 1 | ((hiv or aids) adj6 (patient* or people* or person* or client* or living or men or women or woman or female* or adult* or service* or support* or positiv* or care or caring or affect*)).ti,ab. | 470967 |
| Peer(s), words from subject headings, single word or words from a subject phrase that includes peer(s) | 2 | (peer or peers).hw. | 134476 |
| Words from title or abstract, peer | 3 | peer*.ti,ab. | 332044 |
| Synonyms/related terms for peers | 4 | (lay adj3 (people* or patient* or client*)).ti,ab. | 5993 |
| Synonyms/related terms for peers | 5 | (patient* adj2 expert*).ti,ab. | 7232 |
| **Peers total with synonyms/related** | **6** | **2 or 3 or 4 or 5** | **392228** |
| ***HIV/AIDS and peers*** | ***7*** | ***1 and 6*** | ***8403*** |
| HIV – or AIDS, subject headings | 8 | exp HIV Infections/ or exp Human immunodeficiency virus infection/ or hiv/ | 794523 |
|  | 9 | exp Anti-Retroviral Agents/ or acquired immune deficiency syndrome/ or Acquired Immunodeficiency Syndrome/ or aids/ | 482469 |
| **HIV OR AIDS subject headings** | **10** | **8 or 9** | **917056** |
| Peers – words from title/abstract | 11 | (peer* adj6 (group* or support* or couns* or service* or provide* or care* or mentor* or tutor* or educat* or led)).ti,ab. | 81375 |
| Peers – words form title or abstract or subject headings | 12 | 2 or 11 | 184850 |
| **HIV/AIDS subject headings AND peers (subject/title/abstracts words)** | **13** | **10 and 12** | **6210** |
| **HIV and peers, words from title/abstract or subject headings** | **14** | **7 or 13** | **10836** |
| **Words from title HIV/aids AND peer*** | **15** | **((hiv* or aids) and peer*).ti.** | **1417** |
| **Combined; HIV/AIDS AND peers** | **16** | **14 or 15** | **10968** |
| **Limit year** | **17** | **limit 16 to yr="1981 -Current"** | **10961** |
|  | 18 | limit 17 to yr="1981 - 2013" | 5477 |
|  | *19* | *remove duplicates from 18* | *3222* |
|  | 20 | limit 17 to yr="2014 -Current" | 5484 |
|  | *21* | *remove duplicates from 20* | *3222* |
| **Remove duplicates** | **22** | **21 or 19** | **6444** |

Exporting to EndNote 2000 at a time, [link to segments](https://ovidsp.ovid.com/ovidweb.cgi?T=JS&NEWS=N&PAGE=main&SHAREDSEARCHID=2xo4MM8xtzZrSKjPh6geYExdXbHolXHMO1y5cvB4x4axDwEX3sKtQCh410bLP3X0Q). **Result total 23.05.2021 (search line 16**): EMBASE: 5253, MEDLINE: 3844, APA PsycInfo: 1864: 10961

**CINAHL;Social Work Abstracts;SocINDEX (EBSCOhost), Advanced search, Boolean/Phrase search mode. 23.05.20021**

**Notes:**

- If no field codes, the search is executed in the standard fields, includes words from title, abstract, subject headings
- N# - Near Operator (N) - ex N5 finds the words if they are within five words of one another regardless of the order in which they appear. For example, type tax N5 reform to find results that would match tax reform as well as reform of income tax. N5 = adj6 in Ovid search syntax
- SU = words form subject headings, a single word, or a single word from a subject heading phrase
- TI = words form title
- AB = words from abstract
- MH = exact subject headings used in CINAHL, + includes narrowing terms

|  | **#** | **Query** | **Limiters/Expanders** | **Results** |
| --- | --- | --- | --- | --- |
| People with HIV/AIDS  Words from title or abstract | S1 | TI ((hiv or aids) N5 (patient* or people* or person* or client* or living or men or women or woman or female* or adult* or service* or support* or positiv* or care or caring or affect*)) OR AB ((hiv or aids) N5 (patient* or people* or person* or client* or living or men or women or woman or female* or adult* or service* or support* or positiv* or care or caring or affect*)) |  | 81,647 |
| Peer* words from title, abstract or subject headings | S2 | TI peer* OR AB peer* OR SU peer* |  | 102,895 |
| Synonyms – related peer | S3 | TI (lay N2 (people* or patient* or client*)) OR AB (lay N1 (people* or patient* or client*)) |  | 1,335 |
| Synonyms – related peer | S4 | TI (patient* N1 expert*) OR AB (patient* N1 expert*) |  | 1,443 |
| peers | S5 | S2 OR S3 OR S4 |  | 105,572 |
| *HIV/AIDS and PEERS* | *S6* | *S1 AND S5* |  | *2,221* |
| HIV/AIDS Words from subject headings | S7 | (MH "Acquired Immunodeficiency Syndrome") OR SU aids OR SU hiv OR (MH "Anti-Retroviral Agents+") |  | 149,337 |
| Peer words from subject headings | S8 | SU peer* |  | 31,719 |
| Peers, words from title, abstract, restricted by narrowing words | S9 | TI (peer* N5 (group* or support* or couns* or service* or provide* or care* or mentor* or tutor* or educat* or led)) OR AB (peer* N5 (group* or support* or couns* or service* or provide* or care* or mentor* or tutor* or educat* or led)) |  | 25,470 |
| Peers, subject headings, or title abstract (restricted by narrowing terms) | S10 | S8 OR S9 |  | 49,600 |
| *HIV/aids AND peers (subject) or title/abstract restricted with nearby words* | *S11* | *S7 AND S10* |  | *1,774* |
| *HIV/AIDS AND peers – words from title* | *S12* | *TI (HIV or AIDS) AND TI peer** |  | *527* |
| **Combined HIV/AIDS – and peers** | **S13** | **S6 OR S11 OR S12** |  | **3,065** |
| ***Limit year*** | ***S14*** | ***S6 OR S11 OR S12*** | ***Limiters - Published Date: 19810101-*** | ***3,063***  [***link***](https://tinyurl.com/jvek37cb) |

**Total: 3063;** CINAHL (2,208)**,** SocINDEX (796)**,** Social Work Abstracts (59)
